# Comparing the Effects of Multicomponent and Concurrent Exercise Protocols on Muscle Strength in Older Adults

**DOI:** 10.3390/jfmk9010003

**Published:** 2023-12-20

**Authors:** Filipe Rodrigues, Miguel Jacinto, Raul Antunes, Diogo Monteiro, Diogo Mendes, Rui Matos, Nuno Amaro

**Affiliations:** 1 ESECS—Polytechnic of Leiria, 2411-901 Leiria, Portugal; miguel.s.jacinto@ipleiria.pt (M.J.); raul.antunes@ipleiria.pt (R.A.); diogo.monteiro@ipleiria.pt (D.M.); diogo.l.mendes@ipleiria.pt (D.M.); rui.matos@ipleiria.pt (R.M.); nuno.amaro@ipleiria.pt (N.A.); 2 Life Quality Research Center, 2040-413 Rio Maior, Portugal; 3 Center for Innovative Care and Health Technology, 2410-541 Leiria, Portugal; 4 Research Center in Sport Sciences, Health Sciences and Human Development, 5001-801 Vila Real, Portugal

**Keywords:** healthy aging, fall risk, sarcopenia, exercise program

## Abstract

This study aimed to compare the effects of a multicomponent exercise program and a concurrent exercise program on muscle strength in community-dwelling elderly subjects. Participants (*n* = 35; male = 17; female = 18; Mage = 69.17, SD = 5.01 years) were screened and included in the study. Among them, 19 individuals were assigned to the multicomponent group, while 16 were assigned to the concurrent group. The results of the repeated-measures ANOVA revealed significant main effects for the group factor (F(1,15) = 66.59, *p* < 0.001, η^2^ = 0.81) and the group*time factor (F(1,15) = 16.95, *p* < 0.001, η^2^ = 0.53) for the 30-second chair test. Furthermore, significant main effects were observed only for the group factor (F(1,15) = 19.28, *p* < 0.001, η^2^ = 0.56) for the 30-second arm curl. Regarding the Timed Up and Go test, significant main effects were found for the group factor (F(1,15) = 35.56, *p* < 0.001, η^2^ = 0.70) and the group*time factor (F(1,15) = 11.68, *p* < 0.001, η^2^ = 0.43). Lastly, significant main effects were observed for the group*time factor (F(1,15) = 5.19, *p* = 0.038, η^2^ = 0.25) for handgrip strength. The multicomponent exercise group displayed a greater mean increase compared to the concurrent exercise group. While both the multicomponent and the concurrent exercise programs were effective in improving muscle strength in community-dwelling older adults, the multicomponent exercise group exhibited superior outcomes compared to the concurrent exercise group across the physical fitness measures. These findings suggest that a multicomponent exercise program may be more beneficial for enhancing muscle strength in this population.

## 1. Introduction

The aging population and the promotion of healthy aging have become significant concerns in modern society. With an increasing number of older adults living independently in community settings, it is crucial to identify effective strategies to maintain their physical function and overall wellbeing. Exercise interventions have shown great potential for improving various aspects of health in older adults, particularly muscle strength, which plays a critical role in maintaining independence and quality of life [1]. Implementing targeted exercise programs for this population can lead to substantial benefits in terms of maintaining physical function and preventing age-related decline [2,3].

Community exercise programs play a crucial role in promoting physical activity among community-dwelling older adults. Community exercise programs offer numerous benefits for older adults. First, they provide access to structured physical activity opportunities that are tailored to the needs and abilities of older adults [3,4]. Secondly, these programs typically offer a variety of exercise modalities, allowing participants to choose activities that suit their preferences and interests. Furthermore, community exercise programs foster a sense of belonging and social interaction among older adults [5]. Community exercise programs also offer a level of supervision and guidance from trained professionals, ensuring safe and effective exercise practices [4]. This is particularly important for older adults who may have specific health considerations or limitations. By promoting physical activity among older adults, these programs help reduce the burden of chronic diseases, enhance functional capacity, and potentially decrease healthcare costs [6]. Two prominent approaches in exercise programming for the elderly are multicomponent exercise programs and concurrent exercise programs.

In the realm of exercise programming for the elderly, two prominent paradigms emerge: multicomponent and concurrent exercise programs. The former artfully integrate a variety of exercises—from resistance training to aerobic activities, balance exercises, and flexibility training—aiming to holistically enhance overall health and functional ability [4,7]. In juxtaposition, concurrent exercise programs seamlessly merge aerobic and resistance training within a singular session, with a pronounced focus on amplifying cardiovascular fitness and muscle strength. The significance of muscle strength in the elderly is undeniable, governing their capacity to perform daily tasks, maintain balance, and prevent debilitating falls. Age-associated declines in this strength not only impede routine activities but also escalate the likelihood of falls and the onset of frailty [8]. Nevertheless, when fortified through resistance training, especially when combined with other forms of exercise, these declines can be effectively counteracted, revitalizing debilitated muscles. Both approaches have demonstrated their efficacy in augmenting muscle strength among the elderly; however, their distinct methodologies yield varied results. Multicomponent programs, rich in exercise diversity, promise potentially comprehensive enhancements in overall physical fitness [4,9], whereas concurrent programs, harmonizing cardiovascular and strength training, might edge towards pronounced gains in cardiovascular health and muscle strength [10,11]. The choice between these programs should consider the individual’s specific needs to maximize benefits and support the preservation of muscle strength and overall physical health in the elderly.

While ample evidence underscores the positive impact of exercise on muscle strength in the elderly, a gap exists in comparative studies between multicomponent and concurrent exercise interventions. While previous research has delved into the benefits of one type vis-à-vis a control group, direct comparisons between these two predominant exercise modalities remain sparse. Furthermore, many of the existing exercise protocols emerge from laboratory settings, potentially limiting their applicability to the more dynamic and variable community context. Understanding the relative efficacy of multicomponent versus concurrent exercise programs in the real-world community setting is paramount for sculpting evidence-based exercise guidelines for the elderly [9,12]. Considering this, our study endeavors to juxtapose the effects of multicomponent and concurrent exercise programs on muscle strength among community-dwelling elderly subjects.

We aim to investigate the impact of two exercise programs on muscle strength, and to compare their respective outcomes in the elderly. We hypothesize that both multicomponent and concurrent exercise programs will exert a positive influence on enhancing muscle mass. Furthermore, we postulate that the concurrent exercise program will induce a more substantial increase compared to the multicomponent exercise program, given its inclusion of a more extensive strength training component in the exercise protocol.

## 2. Materials and Methods

### 2.1. Participants

The following eligibility criteria were established for this study based on the objectives and safety considerations: Participants had to be at least 60 years old, capable of standing and walking with or without assistance, not involved in any exercise program, and living in the community. Individuals with a history of chronic neuromuscular, cardiovascular, or metabolic issues that could pose a risk during classes or evaluations were excluded from participation for safety reasons. Moreover, participants were required to be available for all three sessions per week of the physical exercise program and the evaluation periods. Exclusion criteria included participating in less than 75% of the sessions or being absent for more than 5 consecutive sessions. Throughout the study, the participants were advised to maintain their regular physical activity routines, such as engaging in gardening or household activities. The intervention lasted for 20 weeks, with an additional 2 weeks—1 for the pre-intervention assessment and 1 for the post-intervention assessment. The pre- and post-intervention evaluations were conducted by two experienced exercise physiologists, under the supervision of a senior researcher. All of the participants underwent a familiarization session during the pre-intervention assessments. The exercise physiologists provided detailed explanations of all of the tests, and the participants had the opportunity to practice each test without being evaluated for the study. To ensure reliability, intra-observer consistency was examined using the intraclass correlation. The coefficient of 0.88 was derived from the overall mean across all tests for both the multicomponent and concurrent groups. This underscores the reliability of our measurements and provides confidence in the robustness of the data. Any existing health groups or issues related to the intervention were managed in accordance with standard medical practices and documented as adverse events.

In determining the appropriate sample size for our study, an a priori power analysis was conducted using G*Power 3.2. Given the parameters of F tests with repeated-measures ANOVA, and factoring in a within–between interaction, we set an effect size f of 0.25, an alpha error probability of 0.05, and sought a power of 0.95. Additionally, the study design accounted for two groups and two measurements, with a correlation among repeated measures set at 0.8 and a non-sphericity correction epsilon of 1. This analysis recommended a minimum sample size of 24 participants. To account for potential attrition or unforeseen challenges during the study, an additional 10% was factored into our recruitment target, bringing the total to 27 participants.

### 2.2. Procedures

Participants were recruited from the pre-existing community program known as the “60+ Program”, an established educational program for the elderly that incorporates regular physical exercise sessions. Individuals who voluntarily signed up for the 60+ Program expressed their interest in participating in various activities, including research studies. This ensured that the present research was conducted within the framework of the existing educational program tailored for the elderly population. Our recruitment strategies encompassed a variety of channels, including social media platforms, internet advertisements, and the official webpage of the 60+ Program. Detailed information regarding the study, its objectives, and the specific eligibility criteria was disseminated through these avenues. Following an expression of interest, potential participants underwent a screening process to assess their eligibility based on specific criteria, such as being aged 60 years or above and active participation in the 60+ Program. Eligible individuals were provided with comprehensive information about the study procedures, potential risks and benefits, and their rights as research participants. Written informed consent was obtained from all of the participants before their inclusion in the study. To ensure a balanced distribution of participants across the study groups, the allocation of individuals was performed a priori based on their preferences regarding training hour sessions. The participants were not aware in advance of the specific type of training that they would undergo, i.e., multicomponent or concurrent. While gender and age might have naturally varied within these groups due to training hour preferences, our primary objective was to maintain consistency in the allocation process and uphold the blinding to the training type.

The research procedures adhered to the ethical guidelines and principles outlined in the Declaration of Helsinki [13]. The study protocol received approval from the Life Quality Research Center (EA06.2022.CIEQV), guaranteeing the protection of the participants’ rights, privacy, and confidentiality throughout the study. The participants were assured of the voluntary nature of their participation and were given the freedom to withdraw from the study at any time without facing any negative consequences.

### 2.3. Intervention

The exercise interventions in this study adhered to the principles of FITT-VP [14], which encompass frequency, intensity, time, type, volume, and progression. The intervention comprised three weekly morning sessions designed in accordance with the FITT principles and overseen by a highly experienced exercise physiologist specialized in adult and senior exercise prescription. The exercise physiologist provided personalized adaptations, encouragement, and closely monitored exercise intensity using validated measures like the talk test and the Borg scale. Furthermore, after each component, and at the conclusion of each training session, the exercise physiologist distributed these measurement tools to all of the participants. The participants were encouraged to exert themselves according to their individual capabilities. Throughout the intervention period, close monitoring was in place for adherence to the exercise program, progression, and the identification of any adverse events, including incidents such as falls. Exercise sessions were scheduled on weekday mornings, following a one-day-on, one-day-off pattern. The participants took part in three exercise sessions each week, with each session lasting 45−60 min. The intensity of these exercise sessions varied, spanning from low to high levels, and was tailored to each individual’s fitness level. One group followed a multicomponent exercise program, incorporating resistance training, aerobic activities, balance exercises, and flexibility training [4]. The other group participated in a concurrent training program, combining aerobic exercise with resistance training during the same session [11].

The multicomponent program encompassed resistance, cardiorespiratory, balance, agility, and flexibility training. The variety was ensured through three distinct sessions. The exercise sessions began with warm-ups lasting between 5 and 10 min, which included slow walking, dynamic stretching exercises, and dual-task activities. Activities to improve cardiorespiratory fitness, lasting 10−15 min each, were established. These activities encompassed walking, jogging, aerodance, and dance exercises as means to promote cardiorespiratory function. As the name implies, walking entailed moving forward by taking steps on the tips of one’s toes, on heels, and incorporating a knee-raising stride around a predefined path. Jogging, similarly, took place on a predetermined route, with the participants instructed to enhance their hip and knee flexion as they increased their pace. The aerodance routines involved rhythmic exercises synchronized to the music’s beat. Likewise, dance exercises were performed in pairs in accordance with the tempo of the music. A pair of workouts, each spanning a minimum of 10 min, was selected for this purpose. The cardiorespiratory training sessions initially fell within the range of 6 to 7 on the perceived exertion scale, signifying a moderate level of intensity. Subsequently, over the course of 16 weeks, they advanced to achieve a moderate-to-vigorous intensity, as indicated by scores of 7 and 8 on the Borg scale and/or moderate-to-vigorous intensity on the talk test (i.e., heavy breathing, difficulty talking). The resistance training component lasted between 10 and 15 min. Resistance exercises were conducted utilizing various equipment, including bodyweight, ankle weights, rubber bands, and dumbbells. During the circuit, the participants engaged in a range of one to three sets of these resistance exercises. The inter-set rest periods within the circuit varied from 40 to 60 seconds. The chosen exercises specifically targeted essential muscle groups, including those responsible for knee flexion/extension, shoulder abduction/adduction, elbow flexion/extension, pectoral muscles, and back muscles. Each session consisted of four distinct exercises, each aimed at engaging different key muscle groups. In each session, the participants completed chair squats, seated arm abduction and adduction, arm curls, and shoulder shrugs using dumbbells. In another session, the exercises included seated single-leg extension and flexion with ankle weights, arm flexion and extension with dumbbells, and peck deck exercises with rubber bands. During the third session, the participants engaged in standing calf raises, arm curls with a shoulder press, and seated rows using dumbbells. The training intensity transitioned from a light-to-moderate level (4−5 points on the Borg Scale) to a moderate intensity (5−7 points on the Borg Scale) after eight weeks from the commencement of the exercise program, and then to moderate-to-vigorous intensity (7−8 points on the Borg Scale). This adjustment aimed to optimize adaptation and the effectiveness of the workout. The participants initially started with a single set of 8 repetitions and progressively increased to two sets, with each set consisting of 12−15 repetitions. It is worth noting that all of the participants were capable of counting, and if a participant could complete 12 repetitions with minimal effort, the exercise physiologist would encourage an additional 3 repetitions. Balance training, involving static and dynamic elements, was conducted for a duration of 5−10 min, employing wooden sticks, softballs, and balloons. This training encompassed activities such as throwing and/or catching softballs, as well as engaging in single-leg static and dynamic exercises with bats and balloons to enhance balance and agility. To ensure safety during these exercises, the exercise physiologists maintained a safe distance of 2 meters between the participants. Intensity advancement occurred by motivating the participants to either perform exercises at a swifter pace or sustain static movements for extended durations. Consequently, the intensity transitioned from being categorized as light-to-moderate initially (3−5 points on the Borg Scale) to reaching a moderate level (5−7 points on the Borg Scale). It was regulated so as to not exceed moderate intensity, given that the participants had already been involved in moderate-to-vigorous activities during the cardiorespiratory and resistance training segments. Concluding each session, the participants engaged in stretching and flexibility exercises. Every session wrapped up with a 5-min cooldown period focusing on stretching, with an emphasis on maintaining a 1:1 ratio between active and passive stretching. In other words, each stretch was held for 10 seconds, followed by a 10-second pause before moving on to the next exercise or limb. The flexibility routines were customized to address the specific muscles used in the exercises. For example, when the participants performed chair squats, their stretching exercises were focused on the glutes, hamstrings, and quadriceps.

The concurrent program combined elements of both aerobic and resistance training. The exercise sessions commenced with warm-up routines that extended for 5 to 10 min. These warm-ups encompassed activities such as leisurely walking, dynamic stretching exercises, and exercises involving multitasking. Afterwards, for the cardiorespiratory component, much like the multicomponent exercise program, the participants performed activities such as walking, jogging, and aerobic dance exercises. Notably, the cardiorespiratory aspect of this program extended for about 20 min, which was longer in comparison to the multicomponent exercise program. Initially, the cardiorespiratory training sessions were rated at a perceived exertion scale level of 6 to 7, reflecting a moderate level of intensity. However, over the course of 16 weeks, these sessions progressed to attain a moderate-to-vigorous level of intensity, as evidenced by scores of 7 and 8 on the Borg scale and/or the experience of a moderate-to-vigorous level of intensity according to the talk test (i.e., characterized by heavy breathing and difficulty talking). The resistance training sessions, spanning 15−20 min in duration, incorporated identical exercises to those in the multicomponent program. The participants in the concurrent training intervention executed the very same exercises as their counterparts in the multicomponent intervention. However, the distinguishing factor was that in the concurrent training intervention, the participants began with a single set of 8 repetitions and subsequently advanced to three sets, which was one set more than the multicomponent intervention participants. With the inclusion of this additional set for each exercise, the training volume exceeded that of the multicomponent intervention. The training intensity shifted from an initial light-to-moderate level (as indicated by 4−5 points on the Borg Scale) to a moderate intensity (ranging from 5−7 points on the Borg Scale) after eight weeks from the start of the exercise program. Subsequently, it progressed to a moderate-to-vigorous intensity (7−8 points on the Borg Scale). At the end of each session, the participants participated in stretching and flexibility exercises. A 5-min cooldown phase concluded every session, with a strong focus on stretching and a particular emphasis on sustaining a 1:1 balance between active and passive stretching. To clarify, this entailed holding each stretch for 10 seconds, followed by a 10-second pause before proceeding to the next exercise or limb. Flexibility exercises were tailored to the muscles that were engaged in the activities. For instance, if the participants carried out chair squats, they incorporated stretching exercises targeting the glutes, hamstrings, and quadriceps.

### 2.4. Outcomes

The following outcome measures were assessed in all subjects: the 30-second chair stand test, the 30-second arm curl test, the Timed Up and Go (TUG) test, and the handgrip strength test. These measures were selected to evaluate various aspects of physical function and muscle strength in community-dwelling older adults. The 30-second chair stand test assesses lower body strength and functional mobility. The participants were instructed to stand up from a seated position and sit back down as many times as possible within 30 seconds. This test is widely used in assessing lower extremity muscle strength and has demonstrated good validity and reliability in older adults [15]. The 30-second arm curl test evaluates upper body strength and endurance. In this test, the participants were instructed to complete as many bicep curls as they could within a 30-second timeframe using a designated weight (female = 2.5 kg; male = 3.5 kg). The participants used their dominant arm and maintained proper form throughout the test to ensure accurate results. This assessment provides insight into the muscle strength of the upper extremities and has been validated as both a reliable and accurate measure for older adults [15]. The TUG test assesses the time taken by a participant to stand up from a chair, walk 8 feet (2.44 m), turn around, return, and sit back down. The TUG test is widely used as a functional assessment tool in older adults and has demonstrated good reliability [15,16]. Handgrip strength is a widely used measure of overall muscle strength and a predictor of functional performance in older adults. The CAMRY EH101 Electronic Hand Dynamometer (Zhongshan Camry Electronic Co. Ltd., Zhongshan, China) was employed to measure handgrip strength. The participants were instructed to exert maximum effort while seated on a chair with back support and fixed armrests, ensuring that their feet were flat on the floor and their forearms rested on the chair’s arms. The investigator applied a motivational stimulus to encourage the participant to exert their maximum grip effort. Two values were taken in the dominant hand, and the highest value was used for analysis. This test involved the participants squeezing a dynamometer with maximum force using their dominant hand. Handgrip strength has shown strong associations with various health outcomes and is considered to be a valid and reliable measure of muscle strength [17].

### 2.5. Statistical Analyses

Descriptive statistics, including means and standard deviations, were calculated for all variables under investigation. The normality of the data was assessed using the Shapiro–Wilk test for sample sizes less than 50, while homoscedasticity was examined using Levene’s test. To explore differences between dependent variables, a within–between–within ANOVA 2 × 2 design (2 groups × 2 time points) was performed using SPSS version 27. The significance level for rejecting the null hypothesis was set at 5% for all statistical tests. Sphericity assumptions were evaluated using Mauchly’s test, and in cases where this assumption was violated, the Greenhouse–Geisser adjusted values and degrees of freedom were reported, which are indicated by the presence of decimal degrees of freedom. Post hoc tests with Bonferroni adjustments were conducted following the repeated-measures analyses to examine pairwise comparisons. The effect size, η_p_^2^, was calculated, and the reference values for interpretation were as follows: “small” effect = 0.01, “medium” effect = 0.06, and “large” effect = 0.14. All statistical analyses were performed using IBM SPSS Statistics version 27.

Attendance was evaluated in every session by the exercise physiologist before commencing the exercise session. Data from participants were included in the analyses if the participant attended 75% or more of the sessions.

## 3. Results

**Figure 1:**
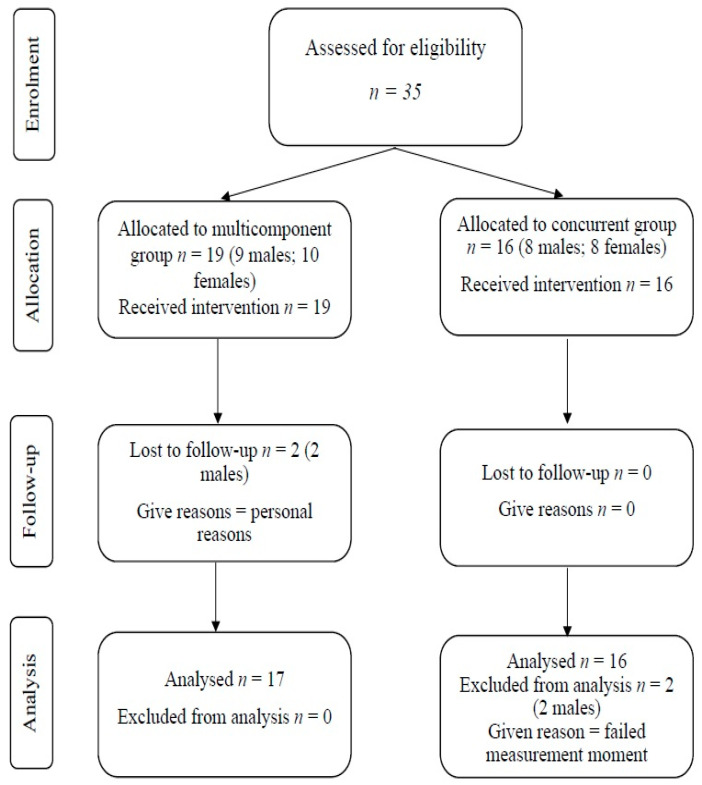
Flowchart of the participants.

A flowchart illustrating the allocation of participants in this study is presented in Figure 1. Initially, all potential participants (*n* = 35; male = 17; female = 18; Mage = 69.17, SD = 5.01 years) underwent screening and were subsequently included in the study. Among them, 19 individuals (male = 9; female = 10; Mage = 70.05, SD = 4.88 years) were assigned to the multicomponent group, while 16 individuals (male = 8; female = 8; Mage = 68.13, SD = 5.12 years) were assigned to the concurrent group. Two participants from the multicomponent group withdrew from the program due to personal reasons, resulting in a final count of *n* = 17 for the multicomponent group. In contrast, all 16 participants in the concurrent group successfully completed the program. However, two participants from the concurrent group did not participate in the post-protocol measurements, leading to a final count of *n* = 14 for the concurrent group. To address missing data, the expectation-maximization method was employed for imputation, enabling the estimation of missing values based on available data for a more comprehensive analysis. Comparative analysis between the raw data and imputed data revealed no significant differences (*p* < 0.05). Thus, the reported results are related to the raw data for transparency.

Table 1 displays the means and standard deviations for the pre (T0) and post (T1) measurements of the outcome variables: 30-second chair stand, 30-second arm curl, Timed Up and Go, and handgrip strength. For the 30-second chair stand, the repeated-measures ANOVA revealed significant main effects for the group factor (Table 2). The multicomponent group exhibited an increase of 21.75%, increasing from an average of 14.15 to 17.22 repetitions, while the concurrent group showed an increase of 7.37%, rising from 15.58 to 16.73 repetitions from pre- to post-intervention. In the case of the 30-second arm curl, significant main effects were observed solely for the group factor. The multicomponent group demonstrated a significant increase of 23.54%, progressing from an average of 19.84 to 24.53 repetitions, whereas the concurrent group experienced an increase of 4.07%, going from 22.37 to 23.28 repetitions between T0 and T1. Regarding the Timed Up and Go test, the repeated-measures ANOVA results unveiled significant main effects for both the group factor and the group*time factor. The multicomponent group displayed a decrease in time of 2.04%, reducing from 4.89 seconds to 4.79 seconds. Conversely, the concurrent group exhibited an improvement of 9.45%, decreasing from 5.08 seconds to 4.60 seconds from pre- to post-measurement. Lastly, for handgrip strength, significant main effects were observed for the group*time factor. The multicomponent group demonstrated a modest rise of 5.98% in strength, increasing from 30.95 kg to 32.80 kg. In contrast, the concurrent group experienced a minor decline of 0.67%, decreasing from 31.12 kg to 30.91 kg between the two timepoints.

An attendance rate of 87% was observed for participants in the multicomponent exercise group, while the concurrent exercise group reported an 84% attendance rate. Regarding falls and fall-related injuries, the exercise physiologists observed two falls during the intervention period, neither resulting in injuries that hindered continued participation. The participants reported no falls outside of the structured fitness program. Notably, no emergencies or hospitalizations were recorded throughout the study period. 

## 4. Discussion

The primary objective of our study was to contrast the effects of a multicomponent exercise regimen against those of a concurrent exercise program on muscle strength in community-dwelling older adults. Our results, underpinned by the repeated-measures ANOVA, unveiled significant main effects for both the group and the group*time factors, particularly evident in the 30-second chair test and the Timed Up and Go test. For the 30-second arm curl, the group difference stood out as the sole significant main effect, whereas, for handgrip strength, the interaction of group and time was the distinguishing factor. Worthy of mention is the distinct variance in the exercise stimuli between the two groups, a foundational tenet of our study design. This variance was not an oversight but a deliberate intention, designed to tease apart the nuanced impacts and benefits of a multifaceted training strategy versus a combined one. Such intentional differences in training protocols are akin to contrasting an experimental group with a control group, emphasizing the distinctive nature of the training methodologies that we examined.

Our findings highlight that the multicomponent group consistently demonstrated a more pronounced mean improvement across the physical fitness metrics in comparison to the concurrent exercise group. This is consistent with prior research conducted among both physically inactive older women [16] and their active counterparts aged 50–75 years [9,17]. These studies echo our observation of the superior advantages offered by multicomponent exercise programs. Theoretical constructs advocate that integrating diverse exercise modalities, each targeting unique facets of fitness, fosters amplified gains in muscle strength [9]. This is attributed to the collective benefits derived from melding resistance training with balance exercises and aerobic activities. The foundation of this method is rooted in the perception that varied exercise modalities stimulate an array of muscle groups and physiological systems, leading to holistic enhancements in both strength and functional capacity [1]. The predominant main effects detected for the multicomponent group in our research reaffirm the positive influence of this approach on muscle strength across diverse metrics.

Diving deeper into the muscular dynamics, the mechanisms underpinning muscle resistance and strength offer valuable insights into the differential outcomes observed between the two exercise regimes. The multicomponent program, with its blend of resistance, aerobic, and balance exercises, arguably imposes a heightened challenge on the musculature. This demand potentially triggers adaptive responses that bolster the muscle’s force-generation capacity [4]. In juxtaposition, concurrent training, although beneficial for cardiovascular health, might encounter obstacles in fully realizing muscle resistance and strength gains. The merged demands of resistance and aerobic exercises in a single session can, at times, create antagonistic muscular adaptations [10,11]. Such interference can potentially curtail muscle hypertrophy and strength development relative to a dedicated resistance training regimen.

Our study was deliberately designed with a pronounced emphasis on strength measures, as evident from the similar duration dedicated to strength training across both the multicomponent and concurrent exercise interventions. This emphasis was underpinned by the understanding that strength outcomes, particularly in the context of our targeted demographic, have considerable implications for overall physical performance, injury prevention, and functional capacity [2,3,18,19]. However, a discerning examination of the two interventions reveals notable differences in the cardiorespiratory components. While both interventions involved walking, jogging, and aerobic dance exercises, the multicomponent exercise intervention allocated 10 to 15 min to these activities, in contrast to the 20 min dedicated to them in the concurrent exercise program. This heightened aerobic exposure in the concurrent program could potentially introduce confounding factors when analyzing strength outcomes, given the known interference effects of concurrent training on strength adaptations. Moreover, the multicomponent intervention introduced unique components of agility and balance, lasting 5 to 10 min. Agility and balance exercises, although often underestimated, play a pivotal role in neuromuscular performance and strength outcomes. They challenge the musculature in dynamic and often unpredictable patterns, necessitating rapid force production and intricate neuromuscular coordination. Such activities engage a broad spectrum of muscle groups, from core stabilizers to peripheral muscles, potentially augmenting strength adaptations [18,20]. Moreover, the balance component, leveraging props like wooden sticks, softballs, and balloons, further emphasized proprioceptive acuity and muscle activation in stabilizing movements. In contrast, the concurrent program, by its inherent design, sought to amalgamate aerobic and strength training more integrally. While this approach has its merits, particularly for enhancing overall physical fitness, it might induce an interference effect where the pronounced aerobic component could attenuate maximal strength gains [1,2,3].

Both interventions, although distinct in their aerobic and agility/balance elements, consistently upheld fundamental principles such as progressive overload, session diversification for ongoing engagement, and dedicated warm-up and cooldown phases. These shared tenets highlight the significance of these principles in exercise programming, regardless of the specific objectives. While the multicomponent exercise program demonstrated superior outcomes, the substantial enhancements in muscle strength observed from the pre- to post-intervention in the concurrent training group should not be overlooked. As documented in previous studies, the concurrent exercise program, blending resistance and aerobic training, has evidenced favorable impacts on muscle strength [11]. The notable gains seen in the concurrent group resonate with this existing literature [11,20,21]. Even though the improvement of effect sizes were comparatively subdued in the concurrent group versus the multicomponent group, it is crucial to acknowledge that concurrent training also holds the potential for bolstering muscle strength in older adults living in the community. When tailoring exercise regimens for this demographic, individual inclinations and practicality must be weighed [22,23]. Some may perceive the concurrent program as more pleasurable or more manageable, factors that can foster greater long-term commitment and participation. Hence, while the outcomes might render the multicomponent program more effective, the concurrent training remains a commendable alternative for enhancing muscle strength among older adults.

Upon closer analysis of the handgrip strength results, we observed a nuanced interaction between time and group, with a statistically significant time*group interaction effect. This interaction suggests that the two groups had divergent trajectories in handgrip strength across the duration of the study. The absence of significant main effects for time and group on handgrip strength, combined with the notable interaction, suggests that the two exercise protocols had different impacts on handgrip strength over time. One could infer that the multicomponent group’s intervention was more effective in improving handgrip strength compared to the concurrent group, which slightly regressed. However, it is essential to consider the potential implications of our chosen dynamometer system on these outcomes [24]. The instrument’s design and its demand on the participants, especially older adults, could have influenced their genuine capacity or willingness to exert maximum force. As such, the observed non-significant change in the concurrent group may not wholly reflect the actual potential changes in handgrip strength due to the exercise intervention. We recommend that future research endeavors consider employing more user-friendly and intuitive dynamometers, especially when working with older adult populations, to better capture true grip strength without confounding influences [20]. While our study did not explicitly gauge the comfort levels or solicit feedback from the older adults regarding the user-friendliness of the dynamometer, it is pertinent to acknowledge the broader body of evidence highlighting the importance of instrument comfort in accurate measurements. Various studies and expert feedback have intimated that the design and ergonomics of certain dynamometers might not be ideal for older populations, potentially influencing their ability or willingness to exert maximum force [25,26]. This is especially salient when considering the hand anatomy, potential arthritic conditions, and muscle strength in elderly participants. Discomfort or perceived difficulty in using the tool might inadvertently result in lower grip strength measurements. Therefore, in line with these considerations and our observed results, we advocate for future studies to prioritize the use of more ergonomic and comfortable dynamometers, ensuring that the data acquired genuinely represent the strength capacities of older adults without the interference of external factors [19,25,26].

### Limitations and Agenda for Future Research

Our study primarily emphasized strength measures and, as such, did not include comprehensive assessments of aerobic fitness, potentially overlooking the intertwined effects of strength and aerobic training on overall physical performance. Future studies should consider incorporating both strength and aerobic fitness evaluations to provide a more holistic understanding of training effects, especially when examining protocols that blend different exercise modalities. Our study is subject to several limitations that should be acknowledged. Firstly, the a priori allocation of participants to the multicomponent and concurrent exercise groups may have introduced selection bias and potential confounding factors. A randomized allocation would have enhanced the internal validity of the study by minimizing such biases. Secondly, the small sample size in our study restricts the generalizability of the findings. With a limited number of participants, the statistical power to detect smaller effects is diminished, and the precision of our estimates may be compromised. A larger sample size would have provided more robust results and improved the external validity of the study. Another limitation is the absence of follow-up measures. Without long-term assessments, we are unable to ascertain the durability or sustainability of the observed improvements in muscle strength. Conducting follow-up measurements would have allowed us to evaluate the persistence of the intervention effects and provide insights into the long-term benefits of the exercise programs. The study did not include a control group, which might have provided a clearer benchmark for interpreting the observed differences between the two intervention groups. This omission limits the direct attribution of improvements solely to the interventions. Furthermore, our study focused solely on community-dwelling older adults, which may restrict the generalizability of the findings to other populations, such as institutionalized older adults or those with specific health conditions. Future research should aim to include a more diverse sample to enhance the applicability of the results. Lastly, it is important to acknowledge that our study primarily assessed muscle strength as the outcome measure, while other aspects of physical fitness or functional outcomes were not evaluated. A more comprehensive assessment of various fitness parameters would have provided a more holistic understanding of the effects of the exercise programs.

The practical implications of this study are noteworthy, particularly in the context of community programs for older adults. Implementing multicomponent exercise programs in community settings can offer significant advantages in enhancing muscle strength and promoting overall physical fitness in this population. These programs can be designed to incorporate low-cost materials, making them accessible and affordable for community-based initiatives. By utilizing readily available resources, such as resistance bands or bodyweight exercises, the financial burden associated with implementing exercise programs can be minimized, allowing for broader participation and long-term sustainability. Moreover, community exercise programs provide an excellent platform for social engagement among older adults. By participating in group activities and exercises, older adults can foster social connections, enhance their sense of belonging, and combat social isolation. These social interactions can contribute to overall wellbeing and can have positive effects on mental health, creating a supportive and inclusive environment for the elderly population.

## 5. Conclusions

The findings from our study suggest that both multicomponent and concurrent exercise programs have merits in terms of muscle strength improvements in older adults. Specifically, the multicomponent exercise program exhibited greater outcomes in some measures of muscle strength compared to the concurrent exercise program. Notably, while we observed enhancements in certain aspects of muscle strength, handgrip strength did not exhibit significant between-group differences, emphasizing the need for a nuanced interpretation of the results. The benefits of regular exercise for older adults are vast. Engagement in structured exercise routines has been consistently linked to a multitude of health benefits, including, but not limited to, enhanced muscle strength, improved balance, increased mobility, and augmented overall functional capacity. By integrating regular exercise into their daily routine, older adults can not only fortify their physical health but also foster a sense of community and social interaction—elements that are critical to their holistic wellbeing.

Community-based multicomponent exercise programs, especially those that utilize accessible and low-cost materials, present a feasible approach to bolstering muscle strength and overall physical fitness in community-dwelling older adults. Beyond the evident physical advantages, these programs serve as catalysts for social interaction, underlining their importance for the broader wellbeing of the elderly. Given these insights, there is an evident need to further promote, support, and refine community exercise initiatives tailored for the older population. Such endeavors will not only directly benefit their physical health but will also positively impact their overall quality of life.

## Figures and Tables

**Table 1:** Descriptive statistics.

Variables	Units	Multicomponent Group				Concurrent Group			
		M_T0_	SD_T0_	M_T1_	SD_T1_	M_T0_	SD_T0_	M_T1_	SD_T1_
30 s chair stand	Repetitions	14.15	5.03	17.22	5.37	15.58	4.52	16.73	4.66
30 s arm curl	Repetitions	19.84	6.39	24.53	5.73	22.37	2.63	23.28	7.33
Timed Up and Go test	Seconds	4.89	0.64	4.79	0.89	5.08	0.46	4.60	0.46
Handgrip strength	Kilograms	30.95	9.39	32.80	10.35	31.12	6.84	30.91	6.88

Notes: M = mean; SD = standard deviation; T0 = pre-intervention; T1 = post-intervention.

**Table 2:** Repeated measurements comparison.

Variables	Mean Square	F	df1	df2	*p*	η^2^*_p_*	Pairwise Comparisons
30 s chair stand							
Time	2.503	0.170	1	15	0.686	0.011	ns
Group	81.732	66.596	1	15	≤0.001	0.816	1 ≠ 2
Time*group	77.931	16.953	1	15	≤0.001	0.531	1 ≠ 2
30 s arm curl							
Time	51.01	1.31	1	15	0.269	0.081	ns
Group	435.11	19.28	1	15	≤0.001	0.562	1 ≠ 2
Time*group	10.84	0.181	1	15	0.677	0.012	ns
Timed Up and Go test							
Time	0.209	0.593	1	15	0.453	0.038	ns
Group	1.45	35.561	1	15	≤0.001	0.703	1 ≠ 2
Time*group	2.14	11.687	1	15	0.004	0.438	1 ≠ 2
Handgrip strength							
Time	1.683	0.027	1	15	0.871	0.002	ns
Group	17.621	2.62	1	15	0.126	0.149	ns
Time*group	101.635	5.19	1	15	0.038	0.257	1 ≠ 2

Notes: F = F test; df1 and df2 = degrees of freedom; *p* = significance level; η^2^*_p_* = partial eta squared; ns = not significant.

## Data Availability

The data utilized in this study were obtained under a specific license exclusively for the purposes of this research. The data supporting the findings of this study are not publicly available but can be requested and accessed upon reasonable inquiry, subject to permission from the Life Quality Research Center and the corresponding author.
